# Whole-Retina Reduced Electrophysiological Activity in Mice Bearing Retina-Specific Deletion of Vesicular Acetylcholine Transporter

**DOI:** 10.1371/journal.pone.0133989

**Published:** 2015-07-30

**Authors:** Jake Bedore, Amanda C. Martyn, Anson K. C. Li, Eric A. Dolinar, Ian S. McDonald, Stuart G. Coupland, Vania F. Prado, Marco A. Prado, Kathleen A. Hill

**Affiliations:** 1 Department of Biology, The University of Western Ontario, London, Ontario, Canada N6A 5B7; 2 Molecular Medicine, Robarts Research Institute, Schulich School of Medicine & Dentistry, The University of Western Ontario, London, Ontario, Canada N6A 5B7; 3 Ophthalmology, Cellular and Molecular Medicine, University of Ottawa, Ottawa Eye Institute, Ottawa, Ontario, Canada K1H 8L6; The University of Melbourne, AUSTRALIA

## Abstract

**Background:**

Despite rigorous characterization of the role of acetylcholine in retinal development, long-term effects of its absence as a neurotransmitter are unknown. One of the unanswered questions is how acetylcholine contributes to the functional capacity of mature retinal circuits. The current study investigates the effects of disrupting cholinergic signalling in mice, through deletion of vesicular acetylcholine transporter (*VAChT*) in the developing retina, pigmented epithelium, optic nerve and optic stalk, on electrophysiology and structure of the mature retina.

**Methods & Results:**

A combination of electroretinography, optical coherence tomography imaging and histological evaluation assessed retinal integrity in mice bearing retina- targeted (embryonic day 12.5) deletion of VAChT (VAChT^Six3-Cre-flox/flox^) and littermate controls at 5 and 12 months of age. VAChT^Six3-Cre-flox/flox^ mice did not show any gross changes in nuclear layer cellularity or synaptic layer thickness. However, VAChT^Six3-Cre-flox/flox^ mice showed reduced electrophysiological response of the retina to light stimulus under scotopic conditions at 5 and 12 months of age, including reduced a-wave, b-wave, and oscillatory potential (OP) amplitudes and decreased OP peak power and total energy. Reduced a-wave amplitude was proportional to the reduction in b-wave amplitude and not associated with altered a-wave 10%-90% rise time or inner and outer segment thicknesses.

**Significance:**

This study used a novel genetic model in the first examination of function and structure of the mature mouse retina with disruption of cholinergic signalling. Reduced amplitude across the electroretinogram wave form does not suggest dysfunction in specific retinal cell types and could reflect underlying changes in the retinal and/or extraretinal microenvironment. Our findings suggest that release of acetylcholine by VAChT is essential for the normal electrophysiological response of the mature mouse retina.

## Introduction

Development of the mammalian retina involves the sequential assembly and disassembly of a series of transient circuits that are required for patterning of the complex neural networks that ultimately form the adult retina. During early retinal development, these transient circuits between retinal neurons generate spontaneous synchronous activity called retinal waves that propagate across the retina in a synergistic fashion, promoting retinal development [1]. Gap junctions mediate retinal waves prenatally (E16-P0) [2,3]. Postnatal retinal waves are at first mediated by acetylcholine (P0 to P11), followed by glutamatergic transmission (P11 to P14) [4]. Although the importance of cholinergic waves in retinal development is well characterized [5–8] much remains to be understood about how cholinergic signalling contributes to the functional capacity of mature retinal circuits.

Acetylcholine-mediated retinal waves are driven by reciprocal cholinergic connections between starburst amacrine cells (SACs), the only acetylcholine-releasing cell type present in the retina [9], that form on either side of the nascent inner plexiform layer (IPL) of the retina [4]. These retinal waves refine topographical neural maps and convey retinal organization to circuits throughout the visual system even prior to the incipience of vision [6]. The propagation of rhythmic cholinergic retinal waves also plays an integral role in regulating cellular proliferation and differentiation in retinal neurons born during their propagation [10]. Cholinergic waves also regulate synaptogenesis by driving structural changes in dendrites of ganglion cells [11]. As shown by the distribution of nicotinic and muscarinic receptors in the macaque retina, many cells (including horizontal cells) are cholinoceptive [12].

The vesicular acetylcholine transporter (VAChT) is a membrane protein whose primary function is to load acetylcholine into presynaptic vesicles, making the neurotransmitter available for release at the presynaptic terminal [13,14]. Proper release of acetylcholine is strongly dependent upon expression of VAChT, in both the peripheral and central nervous systems [15]. Germline deletion of VAChT is lethal, primarily due to blockade of neuromuscular transmission [16]. In the postnatal rat retina, VAChT expression is seen in two distinct bands of the IPL, corresponding to dendrites of displaced and nondisplaced SACs [17]. An ability to remove acetylcholine signalling through control of VAChT expression prior to the developmentally critical period of cholinergic retinal waves and continuing into maturity enables dissection of the relevance of this signalling on retina structure, electrophysiological activity and the function of the mature retinal neural circuitry.

The visual system of the mouse closely resembles that of humans sufficiently to make the mouse an *in vivo* model suitable for the investigation of vision loss. To determine how the absence of cholinergic signalling affects the structural and functional integrity of the mature mouse retina, we used a conditional VAChT-KO mouse line (VAChT^Six3-Cre-flox/flox^) with targeted deletion of VAChT from forebrain cholinergic neurons and neural retina [18]. VAChT^Six3-Cre-flox/flox^ mice were generated using the Cre-loxP system [18], in which *Cre* recombinase is driven by the *Six3* promoter. By embryonic day (E) 12.5 *Six3* is active in the retina, pigmented epithelium, optic nerve and optic stalk of the eye [19]. This construct effectively eliminates VAChT expression prior to the developmentally critical period of cholinergic signalling (P0-P11) and into adulthood, permitting assessment of the contribution of cholinergic signalling to the complex neural circuitry of the mature retina.

A number of loss-of-function studies using transgenic mouse models have examined the importance of cholinergic signaling on various functional or structural components of the retina [5,7,20–22]. Typically, analysis of the absence of early cholinergic waves is studied during the critical developmental period in which retinal wave activity is occurring within the first two post-natal weeks [4,5,7,8]. Compensatory gap junction-mediated wave propagation has been identified in at least one study [22] but collectively, these studies have not assessed how altered cholinergic signalling affects the electrophysiological function of the mature retina *in vivo*. Long-term effects of aberrant cholinergic signalling in the retina during development and into adulthood are poorly understood. How absence of acetylcholine in the retina influences the function of mature retinal circuits is not completely known [1,5].

Activity of the different retinal cell types can be assessed non-invasively using electroretinography, to measure the gross potential at the corneal surface. The a-wave, b-wave, and oscillatory potentials (OPs) of the electroretinogram (ERG) all represent the responses of distinct cellular structures within the retina. The negatively directed a-wave indicates photoreceptor response to incipient light [23–25]. Under scotopic conditions and dim light stimulation the b-wave is directly generated by bipolar cell depolarization [26–28]. The OPs are composed of four to six high-frequency, low-amplitude wavelets superimposed on the ascending limb of the ERG b-wave [29,30]. The origins of OPs are complex and remain poorly understood, but generally, OPs are initiated by amacrine cells of the inner retina [29]. Early OPs are thought to be associated with photoreceptors and bipolar (interplexiform) cells whereas intermediate and late OPs with the action potential-dependent mechanisms of ganglion cells and amacrine cells [29]. To date, the characteristics of the ERG and the histology of the mature retina with early knockout of cholinergic signalling have not been assessed.

Here, we performed a comprehensive evaluation of eye structure and whole retina electrophysiology in VAChT^Six3-Cre-flox/flox^ adult mice, which included ERG assessment of retinal function *in vivo*, to determine the importance of acetylcholine in mature neural retina integrity. We determined that VAChT is not required for gross organization of retinal layers, but is essential for optimal electrophysiological activity of the mature murine retina.

## Materials and Methods

### Animals

Genetically-modified male mice harbouring a ventral forebrain-specific deletion of VAChT were generated as described previously [18]. Mutant mice (VAChT^Six3-Cre-flox/flox^) and littermate controls (VAChT^flox/flox^) were used in this study. Genotyping was performed as described previously [18]. Mice were housed in standard cages and maintained on a 14/10 hour light/dark cycle, with rodent chow and water available *ad libitum*. The murine retina has matured by two months of age [31]. Therefore, a 5-month-old cohort was first examined; representative of a mature murine retina. A cohort at 12 months of age was also characterized, representative of middle age [32], to determine if further aging correlated with progression or rescue of any observed differences between mutant and control mice. Male mice at 5 months (n = 14 littermate control and n = 7 VAChT^Six3-Cre-flox/flox^) and 12 months (n = 5 littermate control and n = 5 VAChT^Six3-Cre-flox/flox^) were euthanized following *in vivo* assessments of left and right eyes. Gene and protein expression were examined in both genotypes of mice age-matched at 7 to 12 months of age.

### Ethics Statement

All aspects of this study were conducted in accordance with the policies and guidelines set forth by the Canadian Council on Animal Care and were approved by the Animal Use Subcommittee of the University of Western Ontario [Protocol 2009–033; 2008–127], London, ON. All efforts were made to minimize animal suffering.

### qRT-PCR and Western Blotting

Retinal tissue samples were collected and frozen in a mixture of dry ice/ethanol and kept at -80°C until used. Tissues were homogenized with a Kontes Pellet Pestle micro-grinder (Sigma-Aldrich Co., St. Louis, MO, USA), and total RNA was extracted with the Aurum Total RNA Fatty and Fibrous Tissue Pack (Bio-Rad Laboratories (Canada) Ltd., Mississauga, ON, Canada). Quantitative real-time PCR (qRT-PCR) [33] and western blotting [15,34,35] were performed as described previously. Immunoreactive bands were detected using the ECL plus western blotting kit (GE Healthcare, Mississauga ON, Canada). Images were obtained and analyzed using the AlphaImager system (Santa Clara, CA, USA).

### Dark Adaptation and Anesthesia

Electroretinography was performed on ketamine-xylazine anesthetised mice as described previously [36]. Mice were dark-adapted overnight for a minimum of 12 hours based on the rhodopsin regeneration period [37], prior to consecutive *in vivo* ERG, intraocular pressure (IOP) and optical coherence tomography (OCT) testing. Anesthesia and *in vivo* testing were performed in a dark room under red light to ensure continuous dark-adaptation.

### Electroretinography

Pupil dilation was achieved using a single drop of 0.8% Diophenyl-T (Alcon, Mississauga, ON) to each eye. To locally anesthetise both eyes and prevent discomfort caused by contact with instrument electrodes, drops of Alcaine solution (Alcon) were applied topically, along with Tear-Gel (Novartis Pharmaceuticals, Dorval, QC) to hydrate the eye. A gold reference electrode (Grass Technologies, West Warwick, RI) was placed in the mouth of the mouse and a grounding lead (Grass Technologies) was inserted subcutaneously into its tail. Small wire loop electrodes (Grass Technologies) were placed on the cornea after applying natural tear gel (Novartis). Electroretinography was performed for both eyes if impedance did not exceed 5000 Ohms (Ω). A series of pre-programmed white light flashes of 5 μs to 2 ms in duration in eleven steps of increasing stimulus strength (0.001 to 25 cd˖s/m^2^) was emitted from a Colordome stimulator (Diagnosys, Lowell, MA). Given no prior evidence for a selective effect between rods and cones, we used a broad spectrum approach of the scotopic electroretinography that reflects both pure rod at lower stimulus luminances, and mixed rod-cone responses at the higher luminances [38]. If ERG differences were found at the higher stimulus levels, then light adapted ERGs would be recorded. Four results were taken for each flash, and an average of these was used in all subsequent analyses. To ensure scotopic conditions were maintained, the dimmest luminance steps were applied first. For stimulus strengths of 0.001–0.04 cd.s/m^2^ the interstimulus interval was 5 seconds, and for 0.1–25 cd.s/m^2^ levels the interstimulus interval was 10 seconds. Mice were placed on a heating pad to prevent anesthesia-induced hypothermia prior to and between *in vivo* assays.

**Fig 1:**
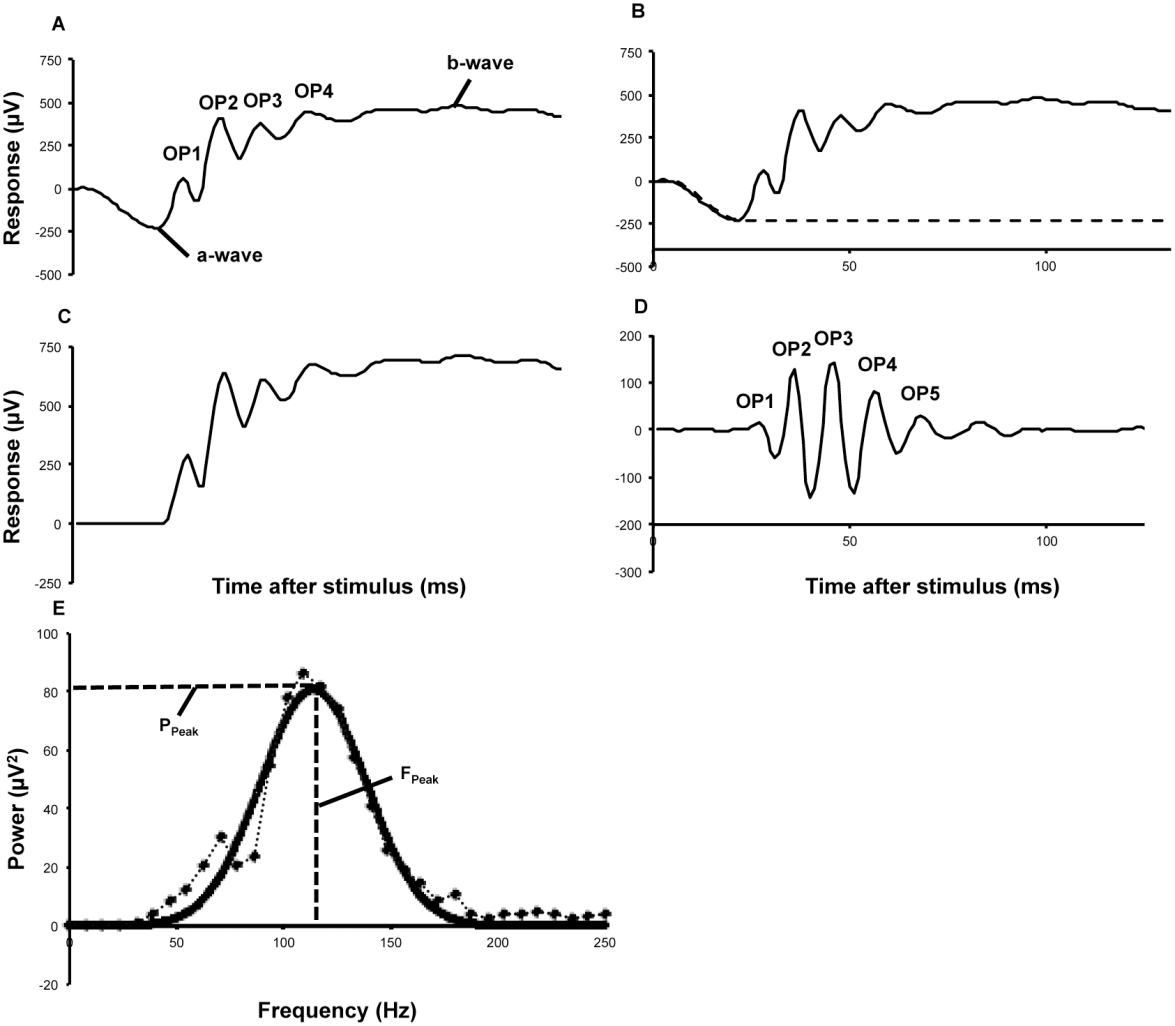
Electroretinogram and OP component modelling. (A) Schematic representation of a trace ERG elicited by a control dark-adapted mouse retina in response to a light stimulus of 25 cd˖s/m^2^ showing major characteristic components of the scotopic ERG. The ERG has 3 components, the a-wave, b-wave, and the oscillatory potentials (OPs). The OPs are the ascending waves between the a- and b-wave. Signal conditioning of OPs from the ERG trace waveform. (B) A digital subtraction of photoreceptor contribution (dashed line) was performed by a mathematical fitting and subtraction of the a-wave from the initial ERG (solid line). (C) The ERG waveform following complete a-wave digital subtraction. (D) The final waveform was passed through a fifth order Butterworth transformation (65–300 Hz) to remove any low or high frequency noise resulting in the final “OP extracted waveform” (solid line). Following waveform extraction, measurements of OP implicit time (ms) and amplitude (μV) were taken for OP2-5. Amplitude of each OP was defined as the difference between the trough and the peak immediately preceding it. Initial implicit time was defined as the time from stimulus to the onset of the OP2 peak. Interpeak distance was the measurement of time (ms) between adjacent peaks (E) Measurement of individual OP features in the frequency-domain. A fast Fourier transform (FFT) was applied to the extracted OP waveform and gave a single-sided smoothed frequency power spectrum (dotted line). The single-sided smoothed frequency power spectrum was fitted to a two-term Gaussian envelope (solid line) and measurements of peak power (P_peak_), peak frequency (F_peak_) and total energy (waveform integration) were taken.

The amplitude of the ERG a-wave was measured from the potential at stimulus onset to the trough of the descending arm and the implicit time was measured as the time from stimulus onset to the inflecting trough (Fig 1A). The ERG a-wave amplitude and implicit time were examined at the three highest luminances only. The amplitude of the b-wave was measured from the trough of the descending arm to the peak of the ascending arm. The implicit time of the b-wave was measured from the stimulus onset to the highest peak of the ascending limb of the ERG. Therefore, implicit times of a- and b- waves were measured from the onset of the light stimulus to the respective a-wave and b-wave negative and positive peaks. To compare the magnitude of a- and b-wave reduction in VAChT^Six3-Cre-flox/flox^ mice, a- and b-wave amplitudes for each VAChT^Six3-Cre-flox/flox^ mouse were divided by their respective age-matched littermate control mean amplitude to produce a ratio representing the proportion of the control amplitude. The a-wave proportion was then plotted against the b-wave proportion for each VAChT^Six3-Cre-flox/flox^ mouse for each of the three highest luminances on a summary plot.

Specific analysis of rod sensitivity was carried out by measuring the rise time of the leading edge of the a-wave as recently described [39] which involves measuring the a-wave peak amplitude along with the measurement of respective times at which the leading edge reaches 10% and 90% of the peak amplitude, heretofore called the 10%-90% rise time. The 10%-90% rise time values were determined at the three highest stimulus strengths of 4 cd˖s/m^2^, 10 cd˖s/m^2^ and 25 cd˖s/m^2^ for both eyes in both VAChT^Six3-Cre-flox/flox^ and littermate controls at both ages.

To extract the OP waveform, the contribution of photoreceptors to the ERG waveform was removed [40–43]. MATLAB software (The MathWorks, Natick, MA) was used to model OPs and digitally subtract the photoreceptor contribution from the overall ERG waveform (Fig 1B); the resulting trace (Fig 1C) was labelled the ‘P2 waveform’. Following the photoreceptor subtraction, the P2 waveform was passed through a fifth order Butterworth filter with a bandpass of 65–300 Hz as previously described [44] to remove extraneous frequencies and residual contamination from the a-wave. This protocol removed both fast and slow PIII [39,45]. The bandwidths that remained comprised the final OP waveform (Fig 1D). OPs were consistently detectable from only the four highest flash intensities, and therefore only these flash intensities were used in OP analysis. To determine whether individual OPs were differentially affected by the loss of VAChT, their amplitudes and implicit times were measured separately. Following the 5^th^ order Butterworth filter, a Fast Fourier Transformation (FFT) was applied to examine OPs in the frequency domain (Fig 1E). The FFT was used to extract the series of sinusoidal waves and graph them as a function of power and frequency [46] using MATLAB software. To avoid powers-of-two problems only the first 128 samples were subjected to the FFT. The frequency-power spectrum was fit to a Gaussian curve to remove artificial spikes in the frequency domain as described previously [47] and measurements of peak frequency and peak power were taken. Total OP energy (total power) was calculated by integrating the area enclosed under the frequency-power spectrum.

### Optical Coherence Tomography

Mice were stabilized and oriented for OCT performed with a Visante instrument (Carl Zeiss Canada Ltd., Toronto, ON) using a custom-designed platform permitting adjustment through the X, Y and Z planes [36]. Diophenyl-T (Alcon) was applied topically to dilate the pupil of both eyes and the instrument was focused at the apex of the corneal surface of each eye prior to image capture. Measurements of central corneal thickness, anterior chamber width, anterior chamber depth, pupil diameter, ratio of pupil diameter to anterior chamber diameter, posterior retina thickness and anterior retina thickness were obtained for each eye using a digital caliper tool (Carl Zeiss Canada Ltd.). Anterior chamber angle of OCT images was measured using ImageJ software (National Institute of Health, Bethesda, Maryland). All measurements for both eyes were taken by two independent scorers blinded to mouse genotype.

### Tonometry

A Tonolab rebound tonometer (Tonolab, Tiolat, Helsinki, Finland) was used to measure IOP of anesthetised mice using a previously established protocol [48]. All readings were taken between 9 AM and 12 PM to control for diurnal variation. Six successive IOP measurements were taken in both eyes of each mouse, between 18 and 22 minutes post-anesthesia. The highest and lowest IOPs for each eye were excluded, and an average was taken of four measures to give one IOP measure per mouse per eye as previously described [48].

### Histology

Enucleated eyes were placed in Telly’s fixative (70% ethanol, 5% formalin, 5% glacial acetic acid) for a minimum of 48 hours and then transferred to 70% ethanol prior to tissue processing. Eyes were processed overnight using a Leica ASP300 (Leica Microsystems, Houston, TX) automated tissue processor (12–16 hours). The processor performed serial one-hour ethanol dehydrations with increasing concentrations of ethanol from 70–100%, followed by three one-hour xylene treatments. Eyes were embedded in paraffin in an orientation that allowed visualization of the central cornea, pupil, central retina and optic nerve (C-cut section). Tissue was sectioned at 6 μm thickness with Leica RM2255 Microtome (Leica Microsystems) and placed on positively charged poly-L-lysine microscope slides (VWR, Mississauga, ON). Sections were stained with hematoxylin and eosin using a Leica Autostainer XL (Leica Microsystems), sealed with Cytoseal Mounting Medium (Richard Allan Scientific, Kalamazoo, MI) and coverslipped (VWR). Eyes were imaged at 20X and 100 using an Arcturus Veritas system (Molecular Devices, Sunnyvale, CA). All histological analyses, including synaptic and nuclear layer thicknesses were performed using previously established protocols [36]. Six measures of the thicknesses of the inner segment (IS) and outer segment (OS) were made 100 μm to the left and 100 μm to the right of the optic nerve head from one eye from at least four mice in each age and genotype cohort. These measures were used to examine the IS:OS ratio with genotype and age. All eyes were examined for neutrophil invasion, retinal-pigmented epithelium disruption, neovascularization and disruption of retinal structure. All measurements were taken by two independent scorers blinded to mouse genotype.

### Statistical Analysis

For all *in vivo* experiments values from left and right eyes were compared using single-factor ANOVA and found not to be significantly different so the average of both was then used all subsequent comparisons. Between-genotype comparisons for ERG measures were made using two-way repeated-measures ANOVA with a Holm-Sidak correction for multiple comparisons. The Sidak’s multiple comparisons test was used to compare VAChT^Six3-Cre-flox/flox^ and littermate controls at each luminance to determine the lowest luminance at which significant differences were detected. Pearson r and coefficient of determination values were also calculated to compare the magnitude of a- and b-wave amplitude reduction in VAChT^Six3-Cre-flox/flox^ mice. A single-factor ANOVA was used in comparisons of OCT data. For histological analyses, the right eye only was assessed. A two-way repeated measures ANOVA was used in analysis of histological measures. A *P* value of 0.05 was used as the threshold of biological significance for all assays. All data were analyzed with Prism 6 software (GraphPad Software, La Jolla, CA).

## Results

### VAChT^Six3-Cre-flox/flox^ mice show reduced VAChT gene and protein expression

**Fig 2:**
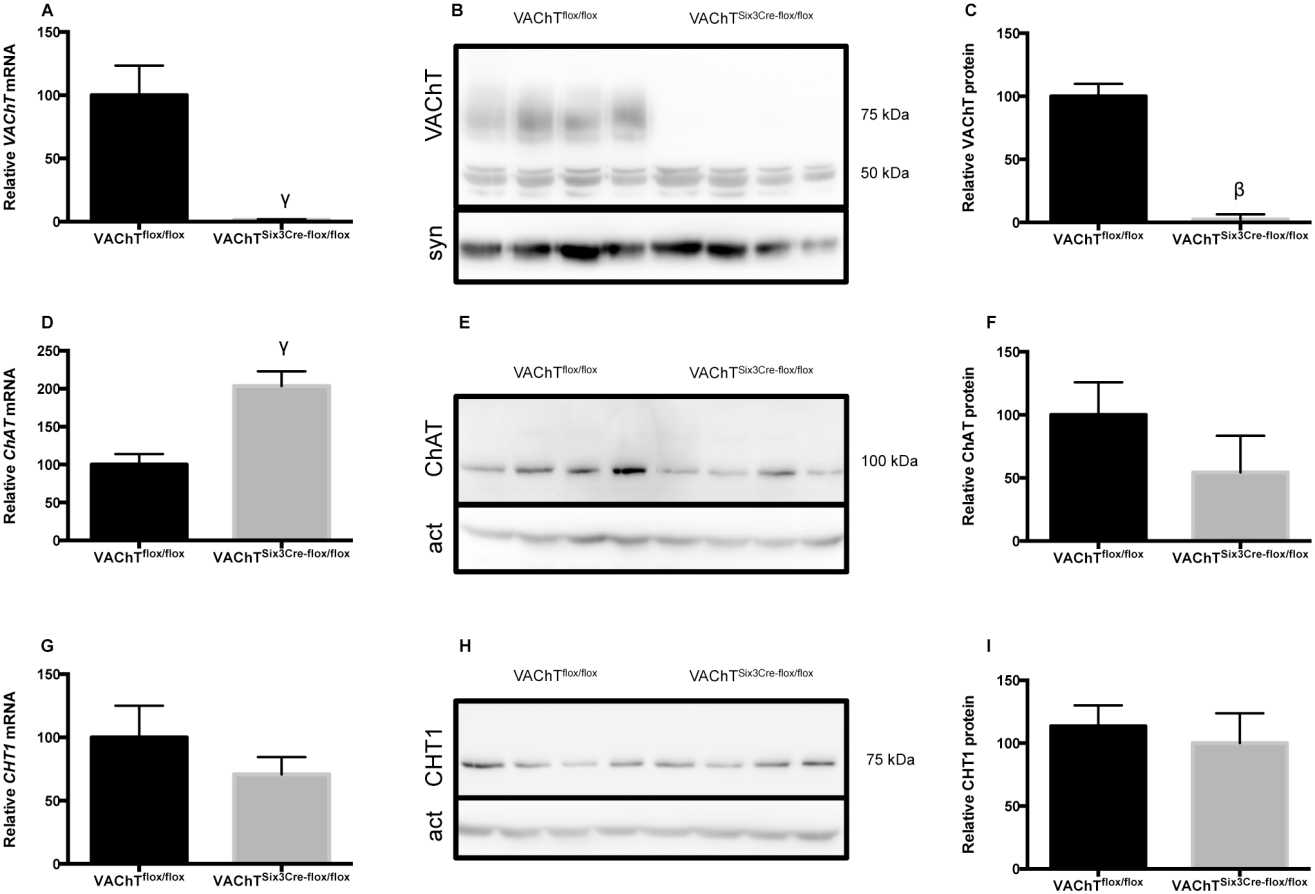
Assessment of gene and protein expression of cholinergic markers. (A, D & G) Quantitative real-time PCR (qRT-PCR) assessed expression levels of VAChT, ChAT and CHT1 mRNA from retinal tissue of VAChT^Six3-Cre-flox/flox^ (n = 5) and littermate control mice (VAChT^flox/flox^, n = 6). (B, E & H) Immunoblotting followed by (C, F & I) densitometry assessed protein levels of cholinergic proteins (A) Expression of *VAChT* mRNA in the retina of VAChT^Six3-Cre-flox/flox^ mice is significantly reduced relative to control. Expression of VAChT protein ChAT increased. (B) Immunoblotting. (B, E, H) Expression levels of VAChT, ChAT and CHT1 protein in the retina of VAChT^Six3-Cre-flox/flox^ and littermate control mice were quantified using densitometry. Expression of VAChT protein in the retina of VAChT^Six3-Cre-flox/flox^ mice (n = 5) is reduced relative to littermate control (n = 6) (C). Synaptophysin protein was used as a loading control for VAChT, while actin protein was used for ChAT and CHT1. Values in (A) and (C) represent mean ± SEM, γ, *P* < 0.01; β, *P* < 0.001 versus control mice.

Real-time PCR (qRT-PCR) and western blotting were used to confirm the deletion of VAChT from the retina of VAChT^Six3-Cre-flox/flox^ mice. qRT-PCR showed that VAChT mRNA is reduced significantly in VAChT^Six3-Cre-flox/flox^ retinas (*P*<0.01; Fig 2A). There is a significant increase in ChAT gene expression (*P*<0.01; Fig 2D), while expression of the high affinity choline transporter CHT1 mRNA is not changed (Fig 2G). Immunoblotting followed by densitometry demonstrated that VAChT protein is significantly reduced in the retinas of VAChT^Six3-Cre-flox/flox^ mice (*P*<0.001; Fig 2B and 2C), while ChAT (Fig 2E and 2F) and CHT1 (Fig 2H and 2I) protein expression are not altered.

### Scotopic electroretinography shows deficits in VAChT^Six3-Cre-flox/flox^ retinas

**Fig 3:**
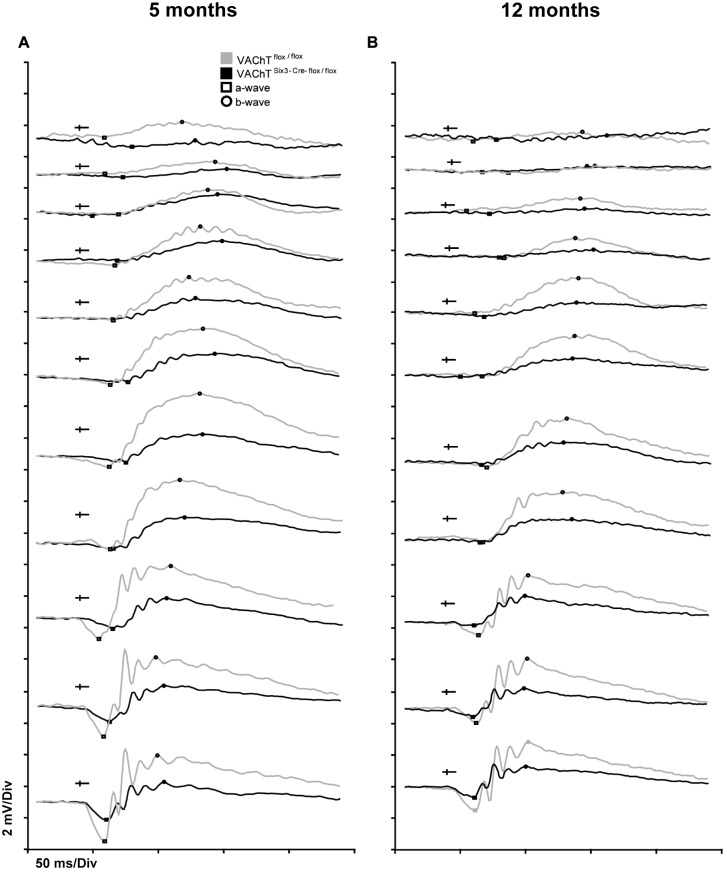
Characterization of retinal electrophysiology under scotopic conditions. Representative scotopic ERG traces for VAChT^Six3-Cre-flox/flox^ and VAChT^flox/flox^ littermate control mice at (A) 5 and (B) 12 months of age at eleven light stimuli of increasing strength. ERG waveforms assessed in response to 11 light stimuli of increasing luminance (0.001 to 25 cd˖s/m^2^). Waveforms are arranged from lowest stimulus luminance (top) to highest (bottom). Amplitudes of the a-wave (squares) and b-wave (circles) are reduced in VAChT^Six3-Cre-flox/flox^ retinas relative to littermate controls, whereas implicit times of the a- and b-waves are unaffected by the loss of *VAChT*.; the stimulus onset is indicated by a sideways cross mark.

**Fig 4:**
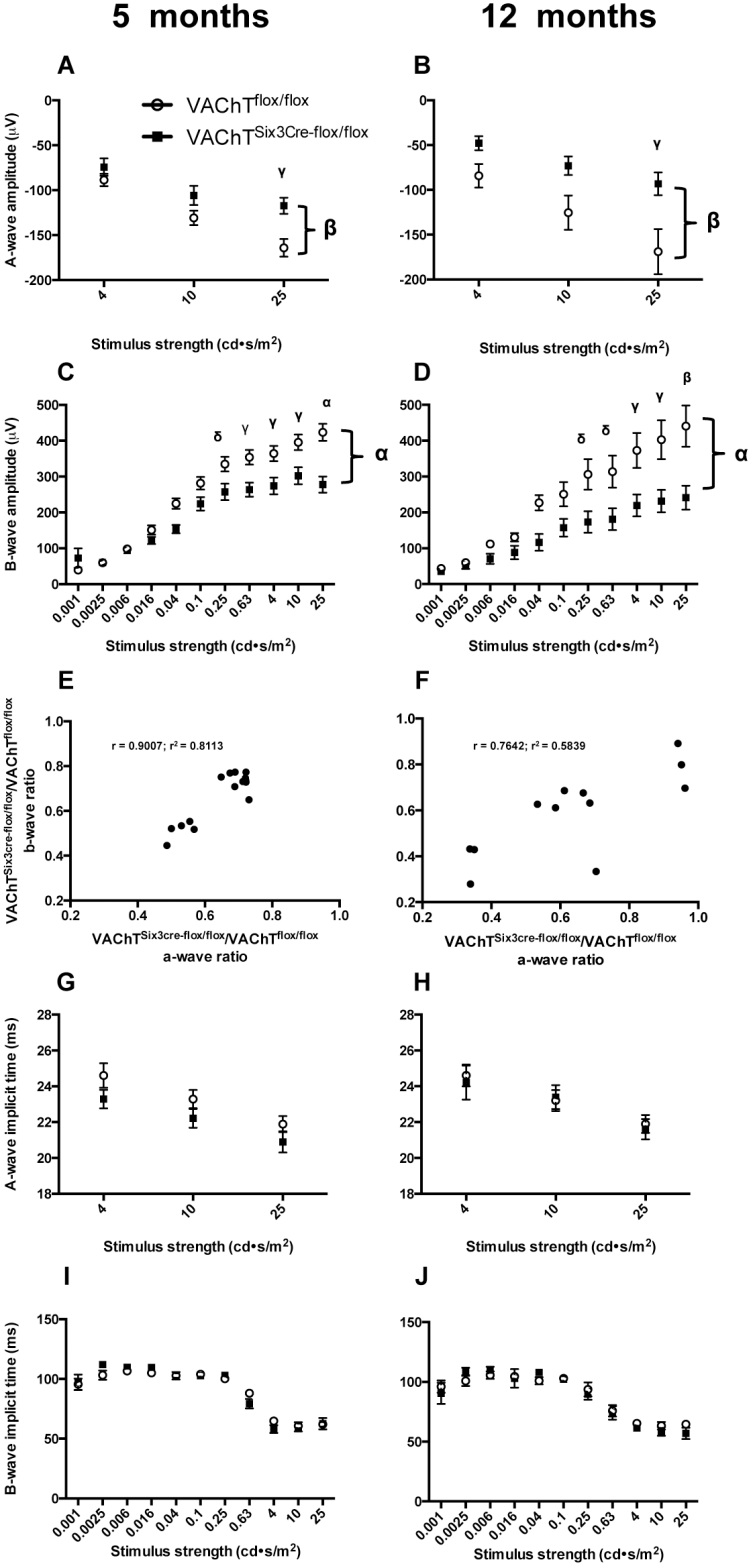
Quantitative assessment of a- and b-waves. Amplitudes of the a-wave (A-B) and b-wave (C-D) are reduced in VAChT^Six3-Cre-flox/flox^ retinas (open circles) relative to littermate controls (VAChT^flox/flox^, closed squares) at 5 (n = 7 VAChT^Six3-Cre-flox/flox^ and n = 14 littermate control) and 12 months of age (n = 5 VAChT^Six3-Cre-flox/flox^ and n = 5 littermate control). The magnitude of a- and b-wave reduction showed a strong correlation at both 5 (E) and 12 months of age (F). Implicit times of the a-wave (G-H) and b-wave (I-J) are unaffected by the absence of *VAChT* at either age examined. Values are the mean ± SEM. For all ERG analyses, large symbols that are located to the far right of each graph and connected to the datasets by brackets indicate significance level of comparisons across luminances by repeated measures two-way ANOVA; smaller symbols above data for individual luminances indicate significance level by Sidak’s multiple comparisons test. δ, *P* < 0.05; γ, *P* < 0.01, β, *P* < 0.001, α, *P* < 0.0001 versus control mice.

VAChT^Six3-Cre-flox/flox^ mice show reduced amplitude of three major components of the scotopic ERG (a-wave, b-wave and OPs) relative to littermate controls over scotopic adapted flash intensities stimulating rod and mixed rod-cone activity [38] at 5 and 12 months of age (Fig 3). VAChT^Six3-Cre-flox/flox^ mice show reduced a-wave amplitude both at 5 months (Fig 4A;, *P*<0.001) and 12 months of age (Fig 4B; *P*<0.001). B-wave amplitude is also significantly decreased in VAChT^Six3-Cre-flox/flox^ mice at 5 months of age (Fig 4C; *P*<0.0001) and 12 months of age (Fig 4D; *P*<0.0001). At 5 months of age, the magnitude of a-wave and b-wave reduction in VAChT^Six3-Cre-flox/flox^ mice showed a strong correlation (Fig 4E; *P*<0.0001; r = 0.9007; r^2^ = 0.8113). At 12 months of age the magnitude of a- and b-wave reduction in VAChT^Six3-Cre-flox/flox^ mice also showed a strong correlation (Fig 4F; *P*<0.01; r = 0.7642; r^2^ = 0.5839). A-wave implicit time is not significantly different from littermate control mice at either 5 or 12 months of age (Fig 4G and 4H). There are also no significant differences in b-wave implicit times between VAChT^Six3-Cre-flox/flox^ and littermate controls at either 5 or 12 months of age (Fig 4I and 4J).

**Fig 5:**
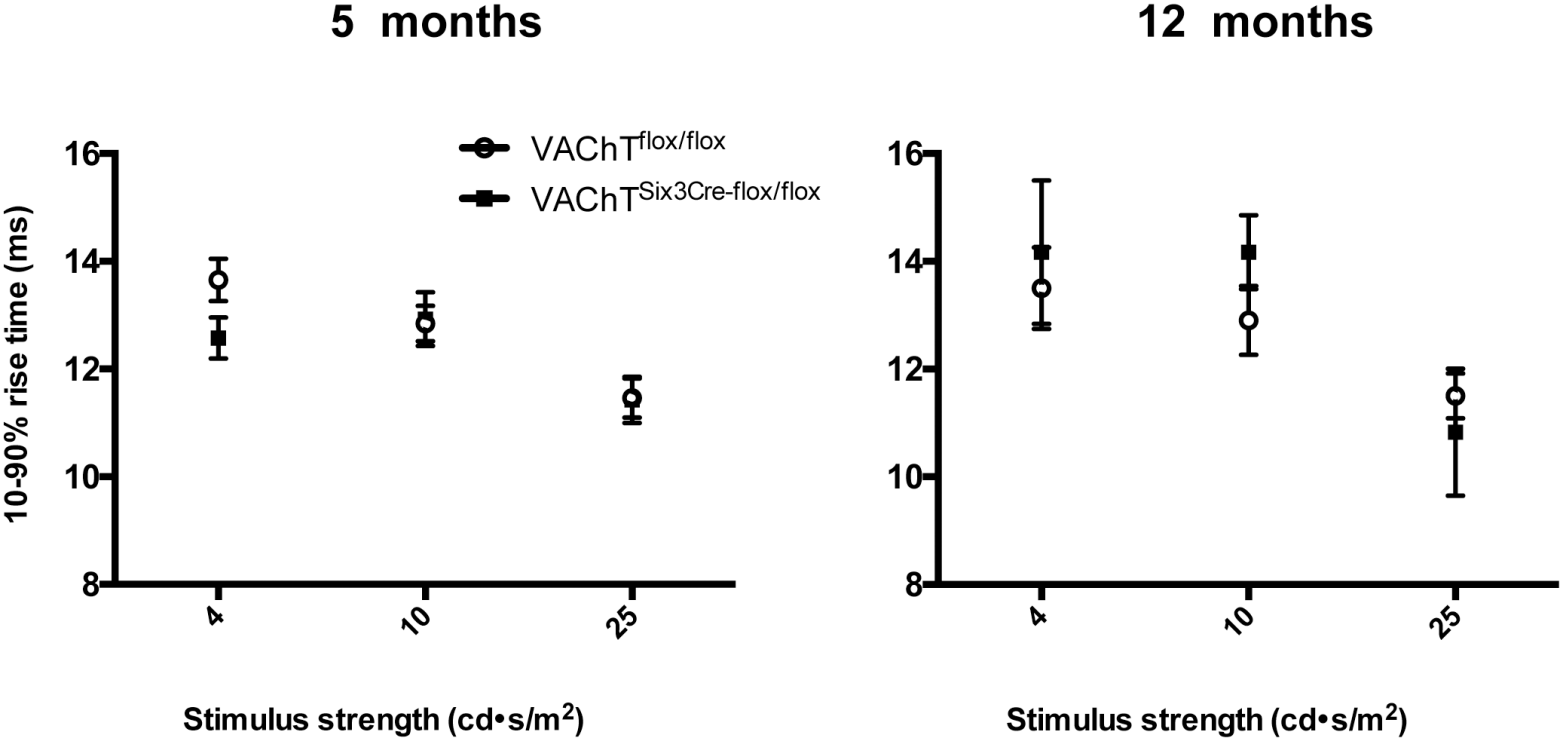
Quantitative assessment of rod sensitivity. The 10%-90% rise time of the leading edge of the a-wave measured for the top three luminances was similar between VAChT^Six3-Cre-flox/flox^ (open circles) and littermate controls (VAChT^flox/flox^, closed squares) at (A) 5 (n = 7 VAChT^Six3-Cre-flox/flox^ and n = 13 littermate control) and (B) 12 months of age (n = 3 VAChT^Six3-Cre-flox/flox^ and n = 5 littermate control). The 10%-90% rise time was unaffected by the absence of *VAChT* at either age examined. Values represent the mean ± SEM.

Specific analysis of rod sensitivity through measurement of the 10% to 90% rise time of the a-wave found no significant differences between VAChT^Six3-Cre-flox/flox^ and littermate controls in 10%-90% rise time at either 5 or 12 months of age (Fig 5). Even at the highest luminance, the 10% to 90% rise time of the a-wave was not different between genotypes. In littermate controls, the shorter 10% to 90% rise time response was as expected for increased luminance (Fig 5). There was no significant difference in the 10% to 90% rise time response with age in littermate control mice.

**Fig 6:**
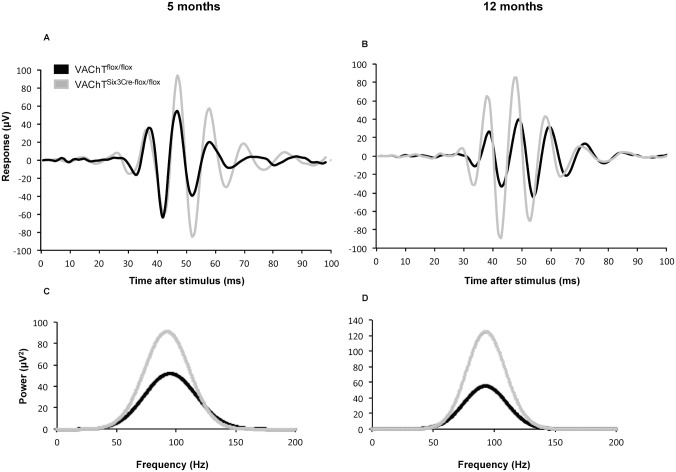
Characterization of OP parameters elicited by VAChT^Six3-Cre-flox/flox^ and control mice under scotopic conditions in response to 10 cd•s/m^2^ light stimulus. (A-B) VAChT^Six3-Cre-flox/flox^ retinas show reductions in individual OP amplitudes in the time-domain representation of ERG OP parameters at (A) 5 and (B) 12 months of age. (C-D) VAChT^Six3-Cre-flox/flox^ retinas also show reductions in OP total energy and power in the frequency-domain representation of ERG OP parameters at (C) 5 and (D) 12 months of age.

**Fig 7:**
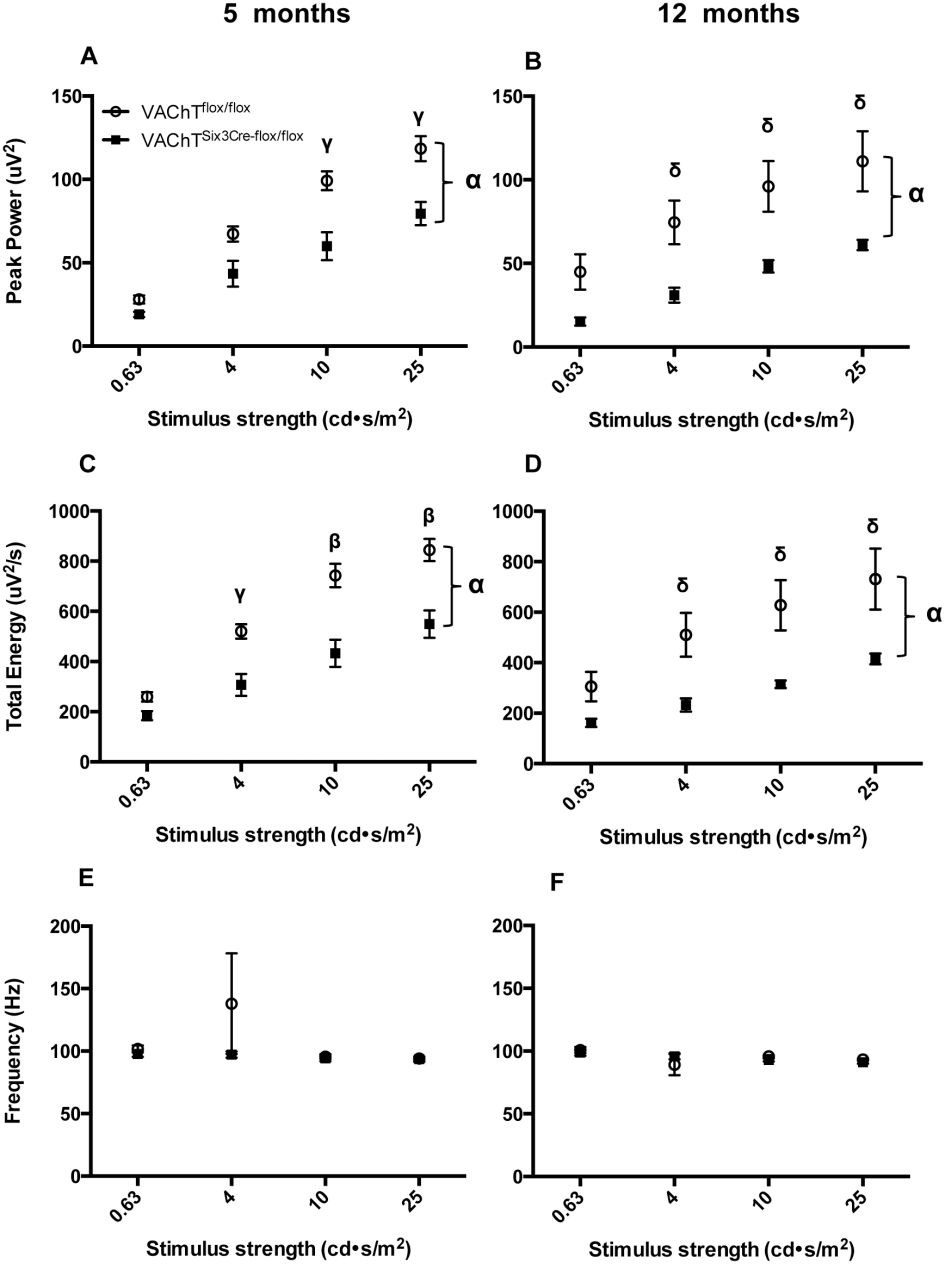
Assessment of oscillatory potential (OP) maximum power, total energy and frequency. A fast Fourier transformation was applied to OP data from the four highest stimulus strengths (0.25–25 cd˖s/m^2^) filtered with a bandpass of 65-300Hz to assess OP profiles in the frequency domain. Peak power is decreased in VAChT^Six3-Cre-flox/flox^ retinas (open circles) relative to littermate controls (closed squares) at (A) 5 (n = 7 VAChT^Six3-Cre-flox/flox^ and n = 12 littermate control) and (B) 12 months of age (n = 5 VAChT^Six3-Cre-flox/flox^ and n = 5 littermate control). Total OP energy is also decreased in VAChT^Six3-Cre-flox/flox^ retinas relative to littermate controls at both (C) 5 and (D) 12 months of age. Average OP frequency does not differ between VAChT^Six3-Cre-flox/flox^ and littermate control mice at either (E) 5 or (F) 12 months of age. Values represent the mean ± SEM. δ, *P* < 0.05; γ, *P* < 0.01, β, *P* < 0.001, α, *P* < 0.0001 versus control mice.

VAChT^Six3-Cre-flox/flox^ mice show changes in OP parameters that are apparent in both the time and frequency domain (Fig 6). The maximum power of the OPs (Fig 7A and 7B; *P* < 0.0001) as well as the total energy of the OPs (Fig 7C and 7D; *P* < 0.0001) are significantly lower in VAChT^Six3-Cre-flox/flox^ than littermate control mice at both ages examined (Fig 7A and 7B; *P* < 0.0001). There are no changes in the dominant frequencies of the OPs between VAChT^Six3-Cre-flox/flox^ mice and littermate controls at either age (Fig 7E and 7F).

**Fig 8:**
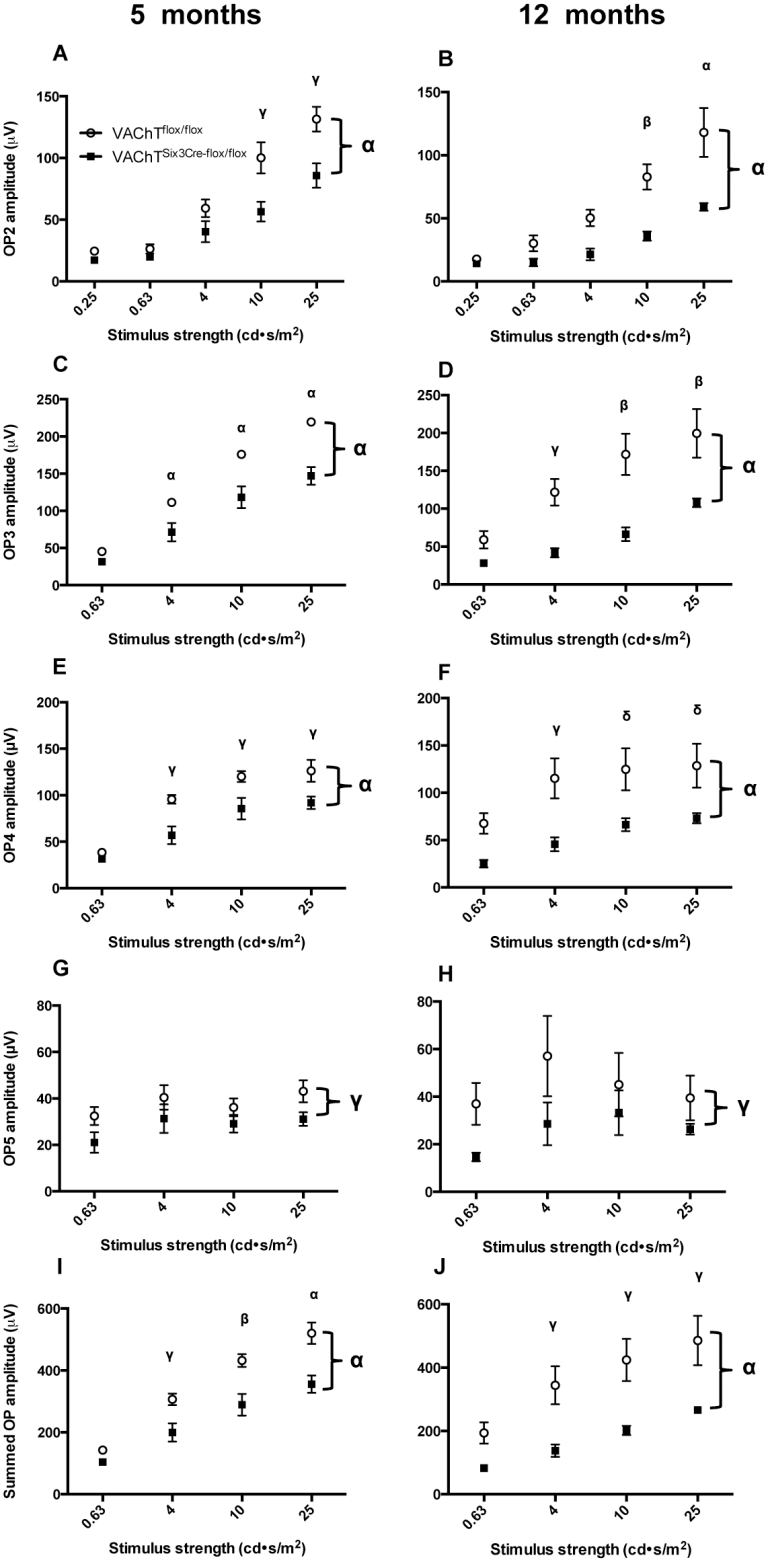
Assessment of oscillatory potential (OP) amplitude profiles. (A-H) Quantification of individual OP amplitudes for the second to fifth OPs (2–5), respectively, also shows a significant reduction in VAChT^Six3-Cre-flox/flox^ mice compared to VAChT^flox/flox^ littermate controls. Quantification of summed OP amplitude for the four OPs measured over the four largest stimulus strengths (0.63–25 cd˖s /m^2^) under scotopic conditions measured shows a significant decrease in VAChT^Six3-Cre-flox/flox^ retinas (open circles) relative to littermate controls (VAChT^flox/flox^, closed squares) at (I) 5 (n = 7 VAChT^Six3-Cre-flox/flox^ and n = 12 littermate control) and (J) 12 months of age (n = 5 VAChT^Six3-Cre-flox/flox^ and n = 5 littermate control). Values represent the mean ± SEM. δ, *P* < 0.05; γ, *P* < 0.01, β, *P* < 0.001, α, *P* < 0.0001 versus control mice.

**Fig 9:**
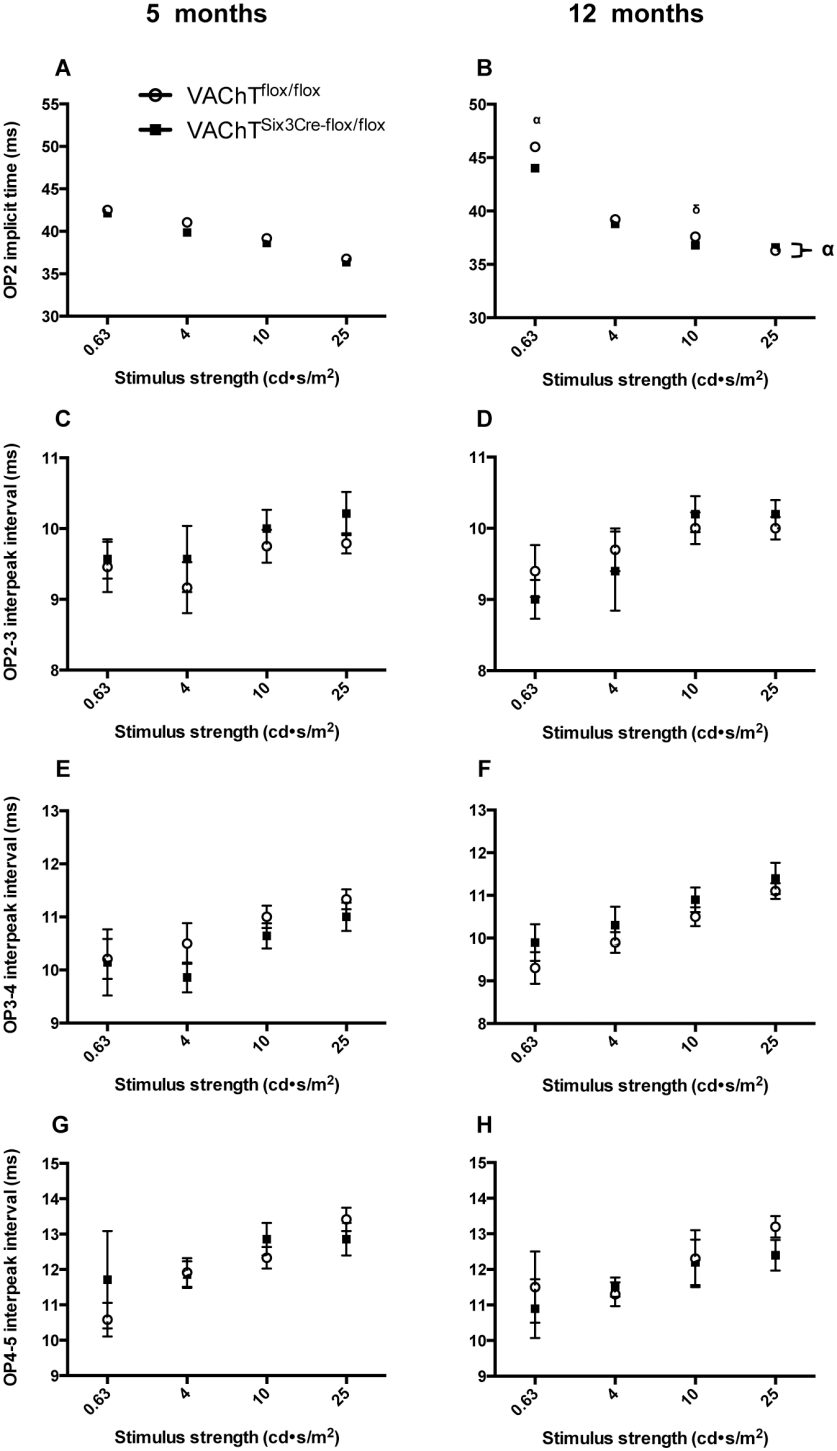
Assessment of oscillatory potential implicit times. Implicit times of OP peaks assessed in VAChT^Six3-Cre-flox/flox^ mice (open circles) and littermate controls (VAChT^flox/flox^, closed squares) at either 5 (VAChT^Six3-Cre-flox/flox^, n = 7; control, n = 12) or 12 months of age (VAChT^Six3-Cre-flox/flox^, n = 5; control, n = 5). The initial implicit time, measures time from stimulus onset to the peak of the first OP, is not different between genotypes at (A) 5 months of age, but is significantly reduced in VAChT^Six3-Cre-flox/flox^ mice at (B) 12 months of age. The implicit times of OP3 (C-D), OP4 (E-F), and OP5 (G-H) are not different between genotypes at either age. δ, *P* < 0.05; α, *P* < 0.0001 versus control mice.

Upon comparing individual OP peaks in the time-domain (Fig 8A–8H) all four OPs are significantly reduced in VAChT^Six3-Cre-flox/flox^ retinas at both ages, OP2 (*P* < 0.0001), OP3 (*P* < 0.0001), OP4 (*P <* 0.0001), OP5 (*P*<0.01). This is also reflected by decreased summed OP amplitude in VAChT^Six3-Cre-flox/flox^ retinas in comparison to littermate control mice at both ages (Fig 8I and 8J; *P* < 0.0001). At 5 months of age, there are no significant differences in OP1 implicit time or any OP interpeak intervals (Fig 9A, 9C, 9E and 9G). However, at 12 months of age, the implicit time of OP1 is significantly shorter in VAChT^Six3-Cre-flox/flox^ mice (*P*<0.0001). The implicit times of all subsequent OPs are not significantly different between genotypes at this age (Fig 9D, 9F and 9H). No significant interactions between age and genotype, indicating worsening or recovery of deficits in VAChT^Six3-Cre-flox/flox^ retinas were found for any of the ERG measures analyzed.

### Histological assessment reveals no structural changes in retinal morphology of VAChT^Six3-Cre-flox/flox^ mice

**Fig 10:**
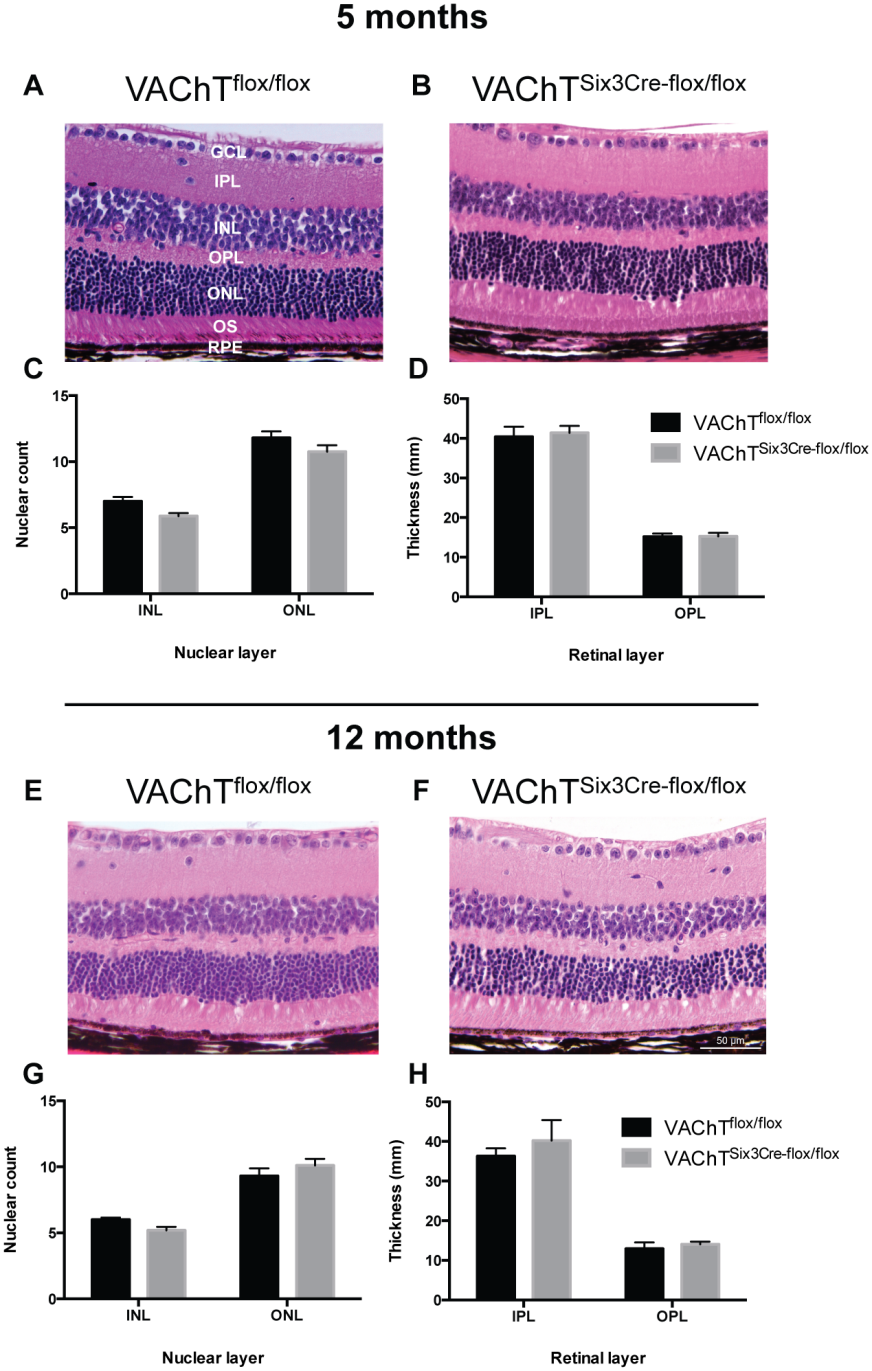
Characterization of retinal layer morphology. (A, B, E & F) Representative images of Hematoxylin and eosin (H&E) stained 6 μ cross sections of retinas from VAChT^flox/flox^ control and VAChT^Six3-Cre-flox/flox^ retinas at (A-B) 5 and (E-F) 12 months of age showing retinal layers. GCL, ganglion cell layer; IPL, inner plexiform layer; INL, inner nuclear layer; OPL, outer plexiform layer; ONL, outer nuclear layer; OS, outer segment; RPE, retinal pigment epithelium. Retinal layer morphology was assessed in VAChT^Six3-Cre-flox/flox^ mice at (C, D) 5 (VAChT^Six3-Cre-flox/flox^, n = 5; littermate control VAChT^flox/flox^, n = 5) and (G-H) 12 months of age (VAChT^Six3-Cre-flox/flox^, n = 5; littermate control, n = 5). INL and ONL cell counts are normal in VAChT^Six3-Cre-flox/flox^ mice at 5 and 12 months of age (C, G). Measurements of synaptic layer thickness of the IPL and OPL show no differences between VAChT^Six3-Cre-flox/flox^ and control mice (D, H). Scale bar represents 50 μm for all images.

To investigate whether structural changes resulted from retina-specific deletion of VAChT, VAChT^Six3-Cre-flox/flox^ and littermate control retinas were assessed histologically at 5 and 12 months of age (Fig 10). Cellularity of the inner and outer nuclear layer is normal in VAChT^Six3-Cre-flox/flox^ mice relative to littermate controls at 5 (Fig 10A) and 12 months of age (Fig 10B). Synaptic layer thicknesses of the outer plexiform layer and inner plexiform layer are not different between VAChT^Six3-Cre-flox/flox^ and littermate control mice (Fig 10C and 10D). The retinal IS:OS ratio did not differ significantly with age or genotype [1.80 ± 0.04 and 1.72 ± 0.04 for 5- and 12-month-old VAChT^Six3-Cre-flox/flox^ mice and 1.69 ± 0.04 and 1.74 ± 0.06 for 5- and 12-month-old littermate control mice (mean+/-SEM)].

### VAChT^Six3-Cre-flox/flox^ mice do not show abnormalities in anterior segment morphology

**Fig 11:**
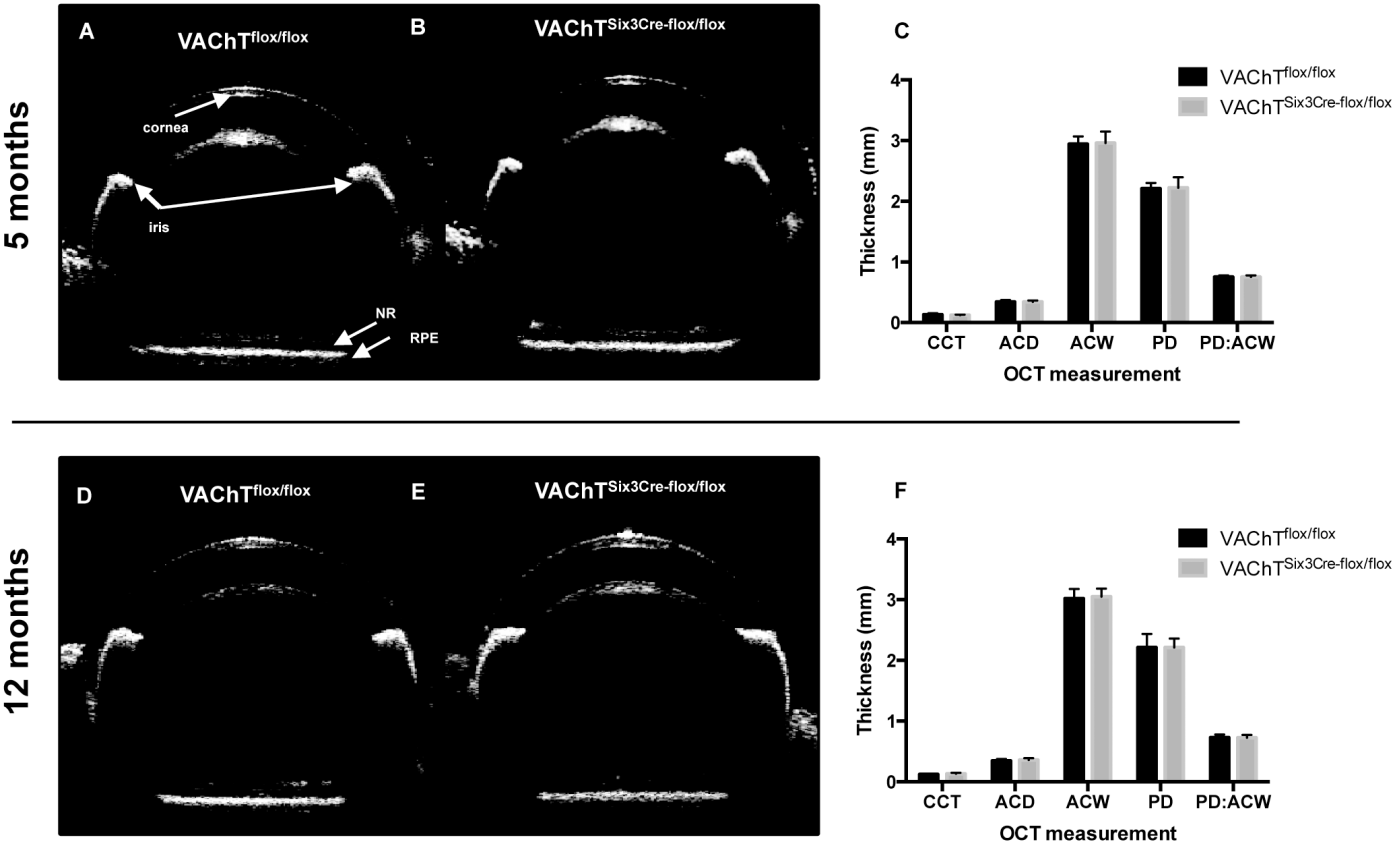
Characterization of anterior segment morphology. OCT images of the anterior segment and retina assessed for morphological changes and the amount of light reaching the retina (A, B, D & E). None of the anterior components of the eye are different between mutant mice and littermate controls at 5 (VAChT^Six3-Cre-flox/flox^, n = 6; control VAChT^flox/flox^, n = 12), or at 12 months of age (VAChT^Six3-Cre-flox/flox^, n = 5; control, n = 5). Images of the anterior segment have been combined with images of the retina. NR, neural retina; RPE, retinal pigment epithelium. (C, F) Central cornea thickness (CCT), anterior chamber diameter (ACD), anterior chamber width (ACW), pupil diameter (PD) and ratio of pupil diameter: anterior chamber depth (PD:ACW) did not differ between VAChT^Six3-Cre-flox/flox^ and VAChT^flox/flox^ mice at 5 or 12 months, respectively.

Three-dimensional *in vivo* imaging of the anterior segment (cornea, lens, iris, and anterior chamber) by OCT reveals no anterior obstructions or abnormalities in VAChT^Six3-Cre-flox/flox^ mice compared to littermate controls, which could impede incipient light from reaching the retina and confound any assessment of principal ERG components (Fig 11A–11D). In addition, no evidence of micropthalmia, anophthalmy, angle closure or cornea or lens opacity that would obstruct light from reaching the retina was found. Central cornea thickness, anterior chamber angle, anterior chamber depth, and thickness of the anterior and posterior retina were quantified using a digiter caliper tool. VAChT^Six3-Cre-flox/flox^ mice show no significant differences from littermate controls in any of these measures taken and at either age (Fig 11E and 11F). Additionally, intraocular pressure (IOP) was assessed to rule out elevated IOP as a contributing factor to any structural aberration, as elevated intraocular pressure (IOP) results in apoptosis-induced retinal ganglion cell death [49]. The IOP (mmHg) of VAChT^Six3-Cre-flox/flox^ mice is not significantly different from littermate controls at either 5 (VAChT^flox/flox^ 8.45 ± 0.42; VAChT^Six3-Cre-flox/flox^ 8.64 ± 0.42) or 12 months of age (VAChT^flox/flox^ 8.07 ± 0.37; VAChT^Six3-Cre-flox/flox^ 8.62 ± 0.19).

## Discussion

Using combined ERG and histological analysis of the mature retina of a new mouse line with targeted deletion of VAChT from the retina, pigmented epithelium, optic nerve and optic stalk, we show that embryonic preclusion of retinal cholinergic signalling leads to reduced electrophysiological response across the retina without compromise of retinal laminar structure. VAChT^Six3-Cre-flox/flox^ mice show decreased amplitudes in major components of their scotopic ERG (a-wave, b-wave and OPs) relative to controls at both 5 and 12 months of age. These mutants also show decreased oscillatory peak power and total power. Thus, in these mutants, loss of cholinergic signalling including cholinergic wave propagation, leads to a deficiency in transmission of visual signals through mature retinal circuits. Reduced a-wave amplitude was proportional to the reduction in b-wave amplitude and not associated with altered a-wave 10%-90% rise time or changes in inner and outer segment thicknesses. Thus, in VAChT mutants, reduced a-wave amplitude was not associated specifically with altered rod sensitivity or rhodopsin abundance suggesting that the observed a-wave reduction may not be associated with deficits in rods specifically. This electrophysiological phenotype in VAChT^Six3-Cre-flox/flox^ retinas did not worsen markedly from 5 to 12 months of age relative to littermate controls, suggesting that VAChT may be important earlier during retinal development but not in maintenance of retinal health.

Genetic mouse models of spatially and temporally disrupted cholinergic signalling have been used to examine the role of cholinergic retinal waves in the development of visual map phenotypes and the structure of the mature visual circuit (reviewed in [5]). Reduced retinal electrophysiological response resulting from absence of cholinergic signalling in VAChT^Six3Cre-flox/flox^ mice is not inconsistent with previous observations of reduced retinal activity and normal retinal structure with some other models of the absence of retinal cholinergic signalling (reviewed in [5]). However, direct comparison of our model and the mature retina ERG with other models of cholinergic signalling is challenging given that these models are used specifically to track the activity and structural development of the retina during the early postnatal, critical period of cholinergic retinal wave propagation.

One of the previous studies showed that deletion of choline acetyltransferase (ChAT), the enzyme responsible for the biosynthesis of acetylcholine, does not result in disruption of retinal waves in the postnatal murine retina, as in the absence of ChAT, gap junctions mediate wave propagation through a compensatory mechanism [22]. We cannot exclude the possibility that such a homeostatic mechanism, which is responsible for maintenance of spontaneous retinal wave activity, is not occurring in VAChT^Six3-Cre-flox/flox^ mice. Retinal waves also occur in the absence of cholinergic signaling in mice lacking neuronal acetylcholine receptor subunit beta-2 (β2 -/- mutants) via gap junctions [7], in contrast to previous reports showing that retinal waves did not occur in these mice [8,50]. However, retinal waves were shown to be aberrant in β2 -/- mutant mice, differing from wild-type in frequency and discharge of the individual cells. It is possible that such a difference might also exist in VAChT mutant mice. β2 -/- mutants also show altered retinal projections into the dorsal lateral geniculate nucleus [7], which may occur in VAChT^Six3-Cre-flox/flox^ mice. However, lateral geniculate nucleus tissue was not assessed in our study. It is also worth noting that there are reports that cell morphology is not altered in other mouse models lacking cholinergic retinal waves [4,51]. Whether impaired retinal function of VAChT^Six3-Cre-flox/flox^ mice is associated with altered signalling or patterning early in development or a consequence of impaired function of mature cholinergic cells would be of interest in future investigations.

Alternate mechanisms for the finding of reduced amplitude over the major ERG waveforms in the presence of normal retinal morphology must also be considered as this has been reported in other models [4,51], one of which [51] found that altered pH levels in the subretinal space of monocarboxylate transporter 3 (*Slc16a8)* knockout mice reduced the saturated a-wave amplitude of the scotopic ERG. Given that *Cre* recombinase activity in VAChT^Six3-Cre-flox/flox^ mice extends to the pigmented epithelium, optic nerve and optic stalk of the eye, extraretinal mechanisms could be responsible for overall dampening of the ERG. Extraretinal mechanisms could include changes in resistivity in RPE or in the vitreous body. Numerous studies investigating the influence of vitrectomy and intravitreous injection of silicone oil have shown significant effect on both a- and b-wave components of the ERG in rabbits [52] and humans [53]. Intravitreal silicone oil appears to have an insulating effect which interferes with the propagation of the ERG. Changes in electrolyte balance within the vitreous humor could account for dampening of the ERG seen in our model. Other investigations using isolated rat retina have shown that retinal function, as measured by the ERG, is affected by oxygen concentration, pH of the incubation media, and ionic concentration of magnesium, sodium, potassium, chloride and calcium [54]. Given that acetylcholine may also act as a vasoactive signal regulating pericyte intracellular CA^2+^ concentration, ionic conductance and contractility with resulting impact on capillary perfusion in the retina [55] the reduced electrophysiological response observed in VAChT^Six3-Cre-flox/flox^ mice may originate within the retina microvasculature. In our model, possible extraretinal factors affecting propagation of the ERG were not investigated should be the subject of future investigation.

During embryonic, fetal and neonatal periods, acetylcholine drives increased intracellular calcium concentration with well-defined spatial and temporal order through M1 muscarinic acetylcholine receptors (mAChRs) in cells of the ventricular zone, and through nicotinic acetylcholine receptors (nAChRs) in ganglion and amacrine cells [56]. Importantly, neurotransmitter-mediated changes in intracellular calcium concentrations can have striking effects on neuronal outgrowth, plasticity and survival [57]. However, VAChT^Six3Cre-flox/flox^ retinas show normal general laminar organization and no gross changes in anatomical organization of retinal interneurons. Similarly, regions devoid of ChAT expression are structurally normal [22].

There is a reasonable hypothesis that calcium signalling in the dendrites of SACs that provides directionally sensitive input to ganglion cells [58] is associated with vesicle-based, calcium-dependent release of acetylcholine. Retinal waves also have proposed roles in organizing the laminar structure of the LGN [59,60], which receives direct synaptic input from directionally sensitive ganglion cells and sharpens retinal directional selectivity [61]. Thus, it is tempting to speculate that loss of VAChT from the retina would result in impaired motion detection in VAChT^Six3-Cre-flox/flox^ mice. However, a more recent study found that directional selectivity is established independent of patterned cholinergic retinal waves corresponding to the firing of starburst amacrine cells [21]. Furthermore, VAChT^Six3-Cre-flox/flox^ mice can perform sophisticated visual tasks of attention and pairwise visual discrimination, with only minor deficits, suggesting that these electrophysiological deficits may be compensated [62]. Future studies could determine whether visual acuity is impaired in VAChT^Six3-Cre-flox/flox^ mice by measuring their optomotor responses [63].

In summary, in this study, we have demonstrated that adult VAChT^Six3-Cre-flox/flox^ mice show reduced electrophysiological response across the retina suggesting that cholinergic signalling in the retina and/or subretinal space is essential for optimal electrophysiological response of the mature mammalian retina. Although much work has been done examining the functional and structural consequences of aberrant cholinergic signalling on retinal structure and function, much remains to be learned, and our research contributes to what is currently known about the importance of cholinergic signalling in visual processing.
